# GCDB: a glaucomatous chemogenomics database for *in silico* drug discovery

**DOI:** 10.1093/database/bay117

**Published:** 2018-10-29

**Authors:** Yu Wei, Jinlong Li, Baiqing Li, Chunfeng Ma, Xuanming Xu, Xu Wang, Aqin Liu, Tengfei Du, Zhonghua Wang, Zhangyong Hong, Jianping Lin

**Affiliations:** 1 State Key Laboratory of Medicinal Chemical Biology, College of Pharmacy and Tianjin Key Laboratory of Molecular Drug Research, Nankai University, Haihe Education Park, 38 Tongyan Road, Tianjin, China; 2 State Key Laboratory of Medicinal Chemical Biology, College of Life Sciences, Nankai University, 94 Weijin Road, Tianjin, China; 3 Biodesign Center, Tianjin Institute of Industrial Biotechnology, Chinese Academy of Sciences, Tianjin, China; 4 Platform of Pharmaceutical Intelligence, Tianjin International Joint Academy of Biomedicine, Tianjin, China

## Abstract

Glaucoma is a group of neurodegenerative diseases that can cause irreversible blindness. The current medications, which mainly reduce intraocular pressure to slow the progression of disease, may have local and systemic side effects. Recently, medications with possible neuroprotective effects have attracted much attention. To assist in the identification of new glaucoma drugs, we created a glaucomatous chemogenomics database (GCDB; http://cadd.pharmacy.nankai.edu.cn/gcdb/home) in which various glaucoma-related chemogenomics data records are assembled, including 275 genes, 105 proteins, 83 approved or clinical trial drugs, 90 206 chemicals associated with 213 093 records of reported bioactivities from 22 324 corresponding bioassays and 5630 references. Moreover, an improved chemical similarity ensemble approach computational algorithm was incorporated in the GCDB to identify new targets and design new drugs. Further, we demonstrated the application of GCDB in a case study screening two chemical libraries, Maybridge and Specs, to identify interactions between small molecules and glaucoma-related proteins. Finally, six and four compounds were selected from the final hits for *in vitro* human glucocorticoid receptor (hGR) and adenosine A3 receptor (A3AR) inhibitory assays, respectively. Of these compounds, six were shown to have inhibitory activities against hGR, with IC_50_ values ranging from 2.92–28.43 μM, whereas one compoundshowed inhibitory activity against A3AR, with an IC_50_ of 6.15 μM. Overall, GCDB will be helpful in target identification and glaucoma chemogenomics data exchange and sharing, and facilitate drug discovery for glaucoma treatment.

## Introduction

Glaucoma, which affects 60–70 million people worldwide, is a painless neurodegenerative disorder and the second cause of blindness after cataracts, resulting in optic nerve damage and irreversible blindness (1–3). The central pathological feature of glaucoma is the permanent loss of vision (4). Glaucoma can be categorized into the following types: namely, open-angle glaucoma and closed-angle glaucoma, according to the iridocorneal angle in the anterior eye. The main risk factor for glaucoma is elevated intraocular pressure (IOP); however, it is not a necessary prerequisite for vision loss because normal tension glaucoma is common (5). High IOP can trigger optic nerve degeneration, retinal ganglion cell (RGC) dysfunction and immune responses (6–8), whereas reduction of IOP can slow vision loss but not necessarily stop the progression of the disease. Currently, to treat glaucoma, the main clinical drugs reduce IOP. These drugs were not developed specifically for glaucoma but rather were originally used to treat other disorders, such as cardiovascular disease (9). Glaucoma is associated with multiple risk factors, including obesity, high blood pressure, migraines, a family history of the disease and pressure in the eye (4). However, the pathogenesis of glaucoma is not yet fully understood. Approximately 10% of glaucoma cases are the result of genetic variants of myocilin, optineurin or WDR36, and 20% of primary open-angle glaucoma patients are involved in an even wider genetic link (10–12). Therefore, the design of novel and effective drugs that are selective to eye tissues to slow the progress of the disease is a challenge in high demand.

With the rapid accumulation of chemical genomics data about glaucoma, it is necessary to manage an increasing number of specific glaucoma targets and ligands effectively for drug research. Several public databases contain almost all glaucoma-associated chemicals, pharmacological, pharmaceutical, targets, pathways and pharmacogenomics in a broad scope of bioinformatics and cheminformatics, such as DrugBank, PubChem and PharmGKB. However, these databases are more analogous to an encyclopaedia than a specific chemogenomics database for drug research. Databases such as PharmGKB specialize in identifying genetic variation in drug response and contains ∼641 drugs, 130 pathways, 100 dosing guidelines and 498 drug labels.The number of drugs and pathways in PharmGKB are far fewer than that in DrugBank and KEGG, containing 9591 drug entries and 525 pathway maps, respectively. ChEMBL focuses on curating bioactive molecules with drug-like properties against drug targets, providing compound screening libraries for lead identification during drug discovery. Here, we cross-reference these data to DrugBank, ChEMBL, KEGG and others. The overall repository of glaucoma-related chemogenomics database (GCDB) covers relationships found between glaucoma-related drugs, chemicals, targets and pathways. We seek to aid researchers in their search for relevant information by sharing glaucoma-specific chemogenomics and providing a powerful computational algorithm. Chemogenomics databases integrated with several computational approaches for estimating a large number of protein–ligand interactions and exploring the multi-target pharmacology across a set of targets, pathways and diseases are commonly used by scientists. *CVDPlatform* (www.cbligand.org/CVD) developed by Zhang *et al.* is a cardiovascular diseases-specific chemogenomics database implemented with chemoinformatics tools for systems pharmacology analysis and predicting molecular mechanisms of anti-cardiovascular agents (13). Kringelum *et al.* developed the ChemProt server, which aims to depict the associations between chemicals and other biological data by exploring the chemogenomics space and linking chemically induced target perturbations to disease *in silico* drug design and discovery (14). To the best of our knowledge, GCDB is the only specific chemogenomics database for drug research concentrating on small molecules that target proteins related to glaucoma. We created an integrated GCDB covering a broad range of genes, chemical structures, approved or clinical trial drugs, affinity values, pathways, bioassays and references. Moreover, an improved chemical similarity ensemble approach (SEA) algorithm developed in our previous study (15) was incorporated in GCDB to predict targets for drugs and identify new scaffolds for glaucoma research. Finally, as a demonstration of the application of GCDB, we identified seven inhibitors that target glaucoma-related proteins [human glucocorticoid receptor (hGR) and adenosine A3 receptor (A3AR)] by combining screens of two commercial libraries, Maybridge and Specs, using GCDB with experimental verifications.

## Materials and methods

### Data sources and collection

The glaucoma-related protein target and chemical data were collected from multiple databases including DrugBank, ClinicalTrial.gov, ChEMBL, BindingDB, KEGG, Pharmacodia, Therapeutic Target Database (TTD), PubMed and SciFinder. For literature from SciFinder and PubMed, the information on glaucoma-related proteins and drugs from experiments were manually extracted. The signalling pathways associated with these targets and drugs were retrieved from KEGG databases. In detail, (i) we typed the keyword ‘glaucoma’ in the search box and clicked ‘search’; (ii) all the entries related to glaucoma from these public data were downloaded; (iii) glaucoma-related research proteins that had been approved, clinical trial and experimental drugs or bioactive compounds were identified; and finally (iv) duplicate proteins from different databases (based on the protein name) were removed to obtain glaucoma-related protein targets. To obtain up-to-date clinical information about glaucoma drugs, we further compared records with related databases, DrugBank, ClinicalTrial.gov, Pharmacodia and TTD by checking manually. Then, approved or clinical trial drugs for glaucoma treatment were stored in the GCDB database. Finally, by browsing the glaucoma-related protein targets from ChEMBL and DrugBank, a list of chemicals, reported bioactivities, bioassays, references and drugs for treatment of other diseases associated with proteins were retrieved. All information including the chemical structures, proteins, pathways, affinity values, bioassays and references, were stored in the backend of GCDB.

### Database implementation

The client side and the RESTful server side of the Glaucoma database were developed using the Angular web framework (http://angular.io) and the Django REST framework (http://www.django-rest-framework.org/), respectively. The database was deployed with Nginx (http://nginx.org/), Gunicorn (http://gunicorn.org/), PostgreSQL (http://postgresql.org/) on an Ubuntu server. In addition, the JSME Javascript plugin (16) was employed to draw structures on the web page, and the RDKit package (http://rdkit.org), a python cheminformatics Toolkit, was utilized for molecular manipulation.

### Web interface

The web interface of GCDB has been designed for various user needs such as the following: (i) keyword or chemical structure search; (ii) data browse; (iii) glaucoma small molecule target prediction.

(i) *Keyword/structure search*. Users can obtain detailed target and chemical information by searching key words including molecular symbol/name/ID, protein/gene symbol and basic pharmacological properties.

GCDB also provides a chemical structural search, including a substructure search and similarity search. The molecular structure can be provided by sketching in the JSME interface or uploading 2D or 3D formats. OpenBabel (17) was used for conversion of the molecules in different chemical data formats.

(ii) *Data browse*. From the browse panel, users can browse all data available in GCDB, including chemical structures, drugs, targets, etc.

(iii) *Target prediction*. By providing chemical structure of interest, users can perform target prediction tasks. After about half a minute of calculation, a list of potential glaucoma-related targets will be displayed together with the *P*-value of each molecule fingerprint method used.

### Target prediction tool

*In silico* target prediction of molecules plays an important role in the process of drug discovery. An improved chemical SEA algorithm developed in our previous study (15) was implemented in GCDB to predict targets for drugs and to identify new scaffolds for glaucoma research. The detailed algorithmic descriptions have been previously published (18). The principle of target prediction by SEA is based on relating proteins using chemical similarity among their ligands. Therefore, SEA predicts new targets for small molecules, toxic probabilities and side effects by exploiting similarity in chemical structures.

### Chemicals and reagents

All 10 tested compounds were purchased from J&K Scientific Ltd. (Shanghai, China).

### Bioassays

Pharmaron (Beijing) was commissioned to perform *in vitro* inhibition screening of hGR and A3AR.

**(1) hGR bioassay.**

The coding sequence of **hGR** ligand-binding domain was inserted into the pBIND expression vector (Promega, E1581) to express GR-GAL4 binding domain chimeric receptors. The expression vector and reporter vector were co-transfected into HEK293T host cells. The principle of the assay is that once the agonist binds to the corresponding GR-GAL4 chimeric receptor, the reporter gene is activated after binding of the chimeric receptor to the GAL4 binding sites. When the antagonist and agonist are in the system, the antagonist will compete with the agonist for binding to the GR-GAL4 chimeric receptor, inhibiting the reporter gene. Cyproterone was used as a positive control. All six test compounds were prepared in dimethyl sulfoxide (DMSO) at 10 mM and further diluted. Each compound was added to wells at final concentrations of 100 μM, 50 μM, 16.67 μM, 5.56 μM, 1.85 μM, 0.62 μM, 0.21 μM, 0.07 μM, 0.02 μM, 0.008 μM and 0.003 μM.

**(2) A3AR cell-based functional assays.**

The four test compounds were evaluated as A3AR antagonists by A3AR-mediated cAMP production in cell-based functional assays. CHO-ADORA3 cells in which the A3AR was transfected in engineered CHO-K1 cells (PerkinElmer) to express the human A_3_AR were employed. The cells were collected by centrifugation and resuspended with Hank’s Buffered Saline Solution, 5 mM 4-(2-hydroxyethyl)-1-piperazineethanesulfonic acid (HEPES), 0.1% bovine serum albumin (BSA) stabilizer (pH 7.4) and 10 μM Rolipram. 2000 cells per well were added to 384 wells solid white plate. Test compounds were prepared with DMSO in 3-fold serial dilutions of 11 concentrations and dispensed to assay plates. Each compound was tested within the range of 1.69 × 10^-3^–100 μM. The serial dilutions of adenosine-5′-N-ethyluronamide (NECA, 10 nl/well) was transferred to 384 wells and incubated at room temperature for 30 min. A LANCE cAMP 384 kit (Perkin Elmer, USA) was used to measure the intracellular cAMP levels. Forskolin (1 μM) was used to stimulate adenylyl cyclase activity and induce cAMP production. NECA and VUF-5574 were used as reference compounds.

### Molecular docking

Compounds were prepared for docking using LigPrep (19). The published A3AR receptor model (PDB 1OEA) and the crystal structure of human GR with antagonist (PDB 3H52) were retrieved from the RCSB Protein Data Bank and used for molecular docking (20). Each protein was prepared using the Schrödinger Protein Preparation Wizard (21). All waters were removed from the PDB files and hydrogen atoms were added. Partial charges and protonation states were assigned. Minimisation of the structures was terminated when the root mean square deviation reached a maximum value of 0.30 Å. Glide standard precision (SP) was used for the docking calculation to estimate protein–ligand binding affinities (22). For hGR, the grid was centred on the ligand within in the protein complex and its inner and outer cavity radii were set as 10 and 25 Å, respectively. The key amino acids in the binding site included Ile559, Ile563, Gln570, Arg611, Phe623, Gln642, Leu753, Glu755 and Pro762. For A3AR, the receptor grid was generated and centred on the putative ligand binding site (23, 24). The key amino acids in the binding site included Leu90, Thr94, Gln167, Phe168, Met172, Val178, Phe182, Ile186, Trp243, Ser247, Asn250 and Tyr254 (25). The other parameters in grid generations and docking were kept at default settings.

## Results and discussion

### GCDB interface

**Figure 1:**
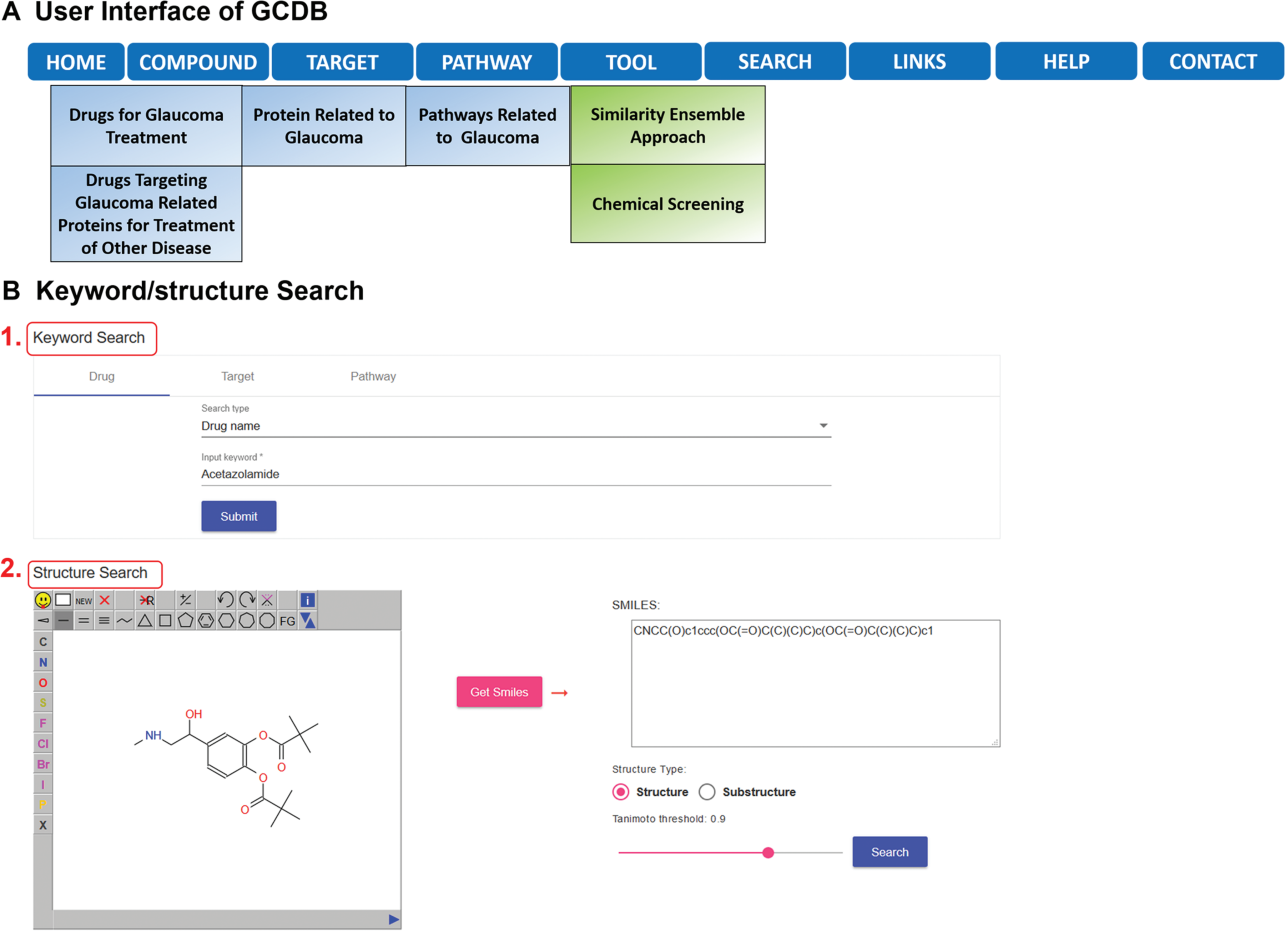
**(A)** Overview of GCDB. **(B)** Keyword/structure Search of GCDB.

GCDB is freely accessible online at http://cadd.pharmacy.nankai.edu.cn/gcdb/home. Using the latest versions of browsers, such as Firefox, Chrome or Internet Explorer, is recommended. GCDB allows users to browse and search the data, but downloading is not supported. However, it provides links to the original databases where users can download desired data from the original databases, such as ChEMBL. As shown in Figure 1A, there are nine pull-down menus: home, compound, target, pathway, search, tool, links, help and contact in the toolbar, and users can browse relevant entries by clicking their sub-menus. Taking ‘COMPOUND’ as an example, users can browse drugs for glaucoma treatment and drugs for treating other diseases that also target glaucoma-related proteins. GCDB enables users to search various keywords, such as drug name and protein name in the ‘Keyword Search’ page (Figure 1B, position 1). Alternatively, users can search a specific structure by sketching it in the ‘Structure Search’ page (Figure 1B, position 2). Users can specify structure types for query, such as substructure search and similarity search.

**Figure 2:**
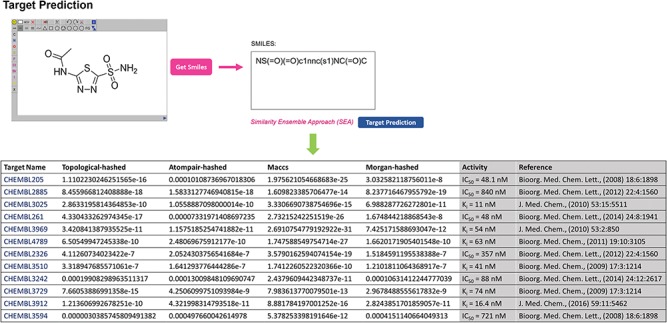
Target prediction page of GCDB and table summarising the results of acetazolamide as an example.

**Figure 3:**
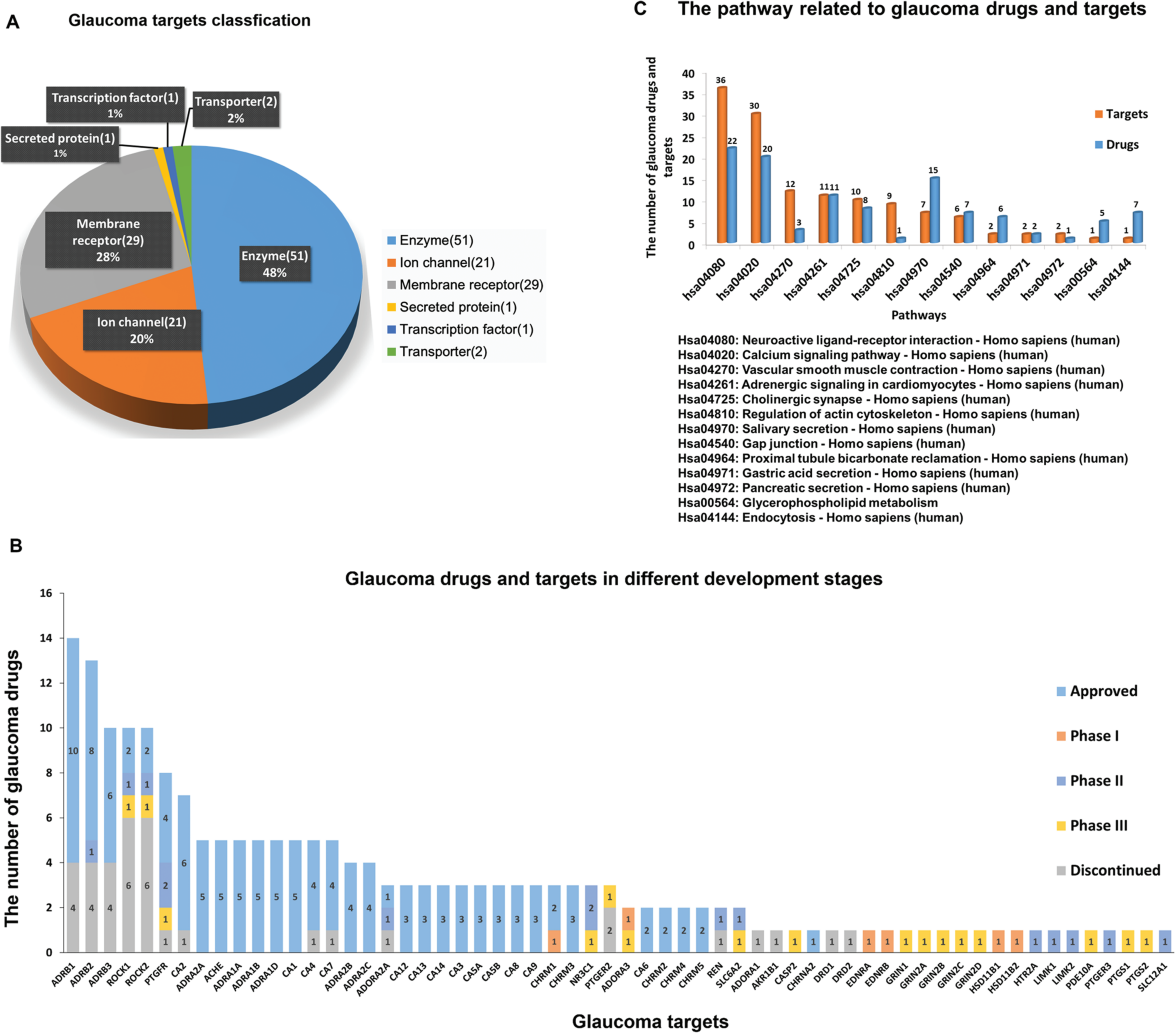
**(A)** The number and variety of glaucoma-related targets. **(B)** Glaucoma drugs in different development phases according to their corresponding targets. **(C)** The pathways in which glaucoma related targets and drugs are involved.

The primary feature of GCDB is its ability to identify the potential drug targets relevant to glaucoma for a given small molecule. ‘Two different tasks, target identification and chemical screening, are currently implemented in the ‘TOOL’ page. If the interactions of a single molecule with proteins need to be explored, users can utilize the SEA online computing algorithm tool to predict possible targets of a query molecule by clicking the ‘Target Identification’ sub-menu. For example, acetazolamide is a carbonic anhydrase inhibitor that is used to treat glaucoma (26). In Target Prediction mode, the input structure of acetazolamide is sketched online or imported in SMILES format as the query. The predictive results obtained directly through SEA are shown in Figure 2. A list of potential protein targets is returned based on the predictive results of a multiple-voting SEA model with four types of molecular fingerprints—topological, atom pair, maccs and morgan (15). The *P*-value is used to indicate the significance of the raw similarity score. Predictions with significance level *P*-values ≤ 0.05 are considered promising targets. The algorithm predicted that acetazolamide is likely to interact with the 12 targets (ChEMBL ID) from the GCDB. These probabilistic targets are shown to be valid in the references (shown in Figure 2). However, ‘Target Identification’ can become inefficient with a large number of molecules. In that case, users can upload a file with multiple molecules (at most 1000) stored in sdf or SMILES formats via ‘Chemical Screening’ sub-menus and provide a valid email address, which is convenient for receiving calculation results. Based on users’ preferences, a single protein can be selected one time in the pull-down menus as the target for virtual screening. After retrieving the users’ job successfully, the calculation starts on the backend server of GCDB. Calculation results with potential target candidates and corresponding *P*-values calculated by SEA in a summary table are returned to users via the provided email address. In addition, GCDB provides users with access to two independent algorithms, ‘PharmMapper’ (27) and ‘ChemMapper’ (28), to derive potential target candidates based on ligand structure predictions in the ‘LINKS’ page. Links to PharmMapper and ChemMapper provide the pharmacophore mapping approach and molecular 3D similarity calculation strategies, respectively, to identify potential target candidates for the given compounds. To access the servers and upload molecule files, users can click on the ‘PharmMapper’ or ‘ChemMapper’ links to the server pages for detailed information.

**Figure 4:**
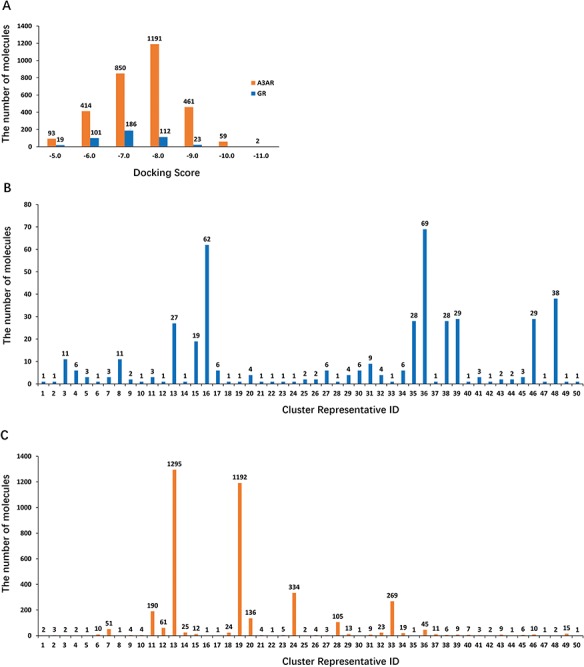
**(A)** Diagram of 3 938 and 447 hits showing docking scores vs number of molecules in each docking score region (orange: A3AR; blue: hGR). **(B)** Diagram of 447 retained hits showing the number of clusters vs the number of molecules in each cluster. **(C)** Diagram of 3938 retained hits showing the number of clusters vs the number of molecules in each cluster.

**Figure 5:**
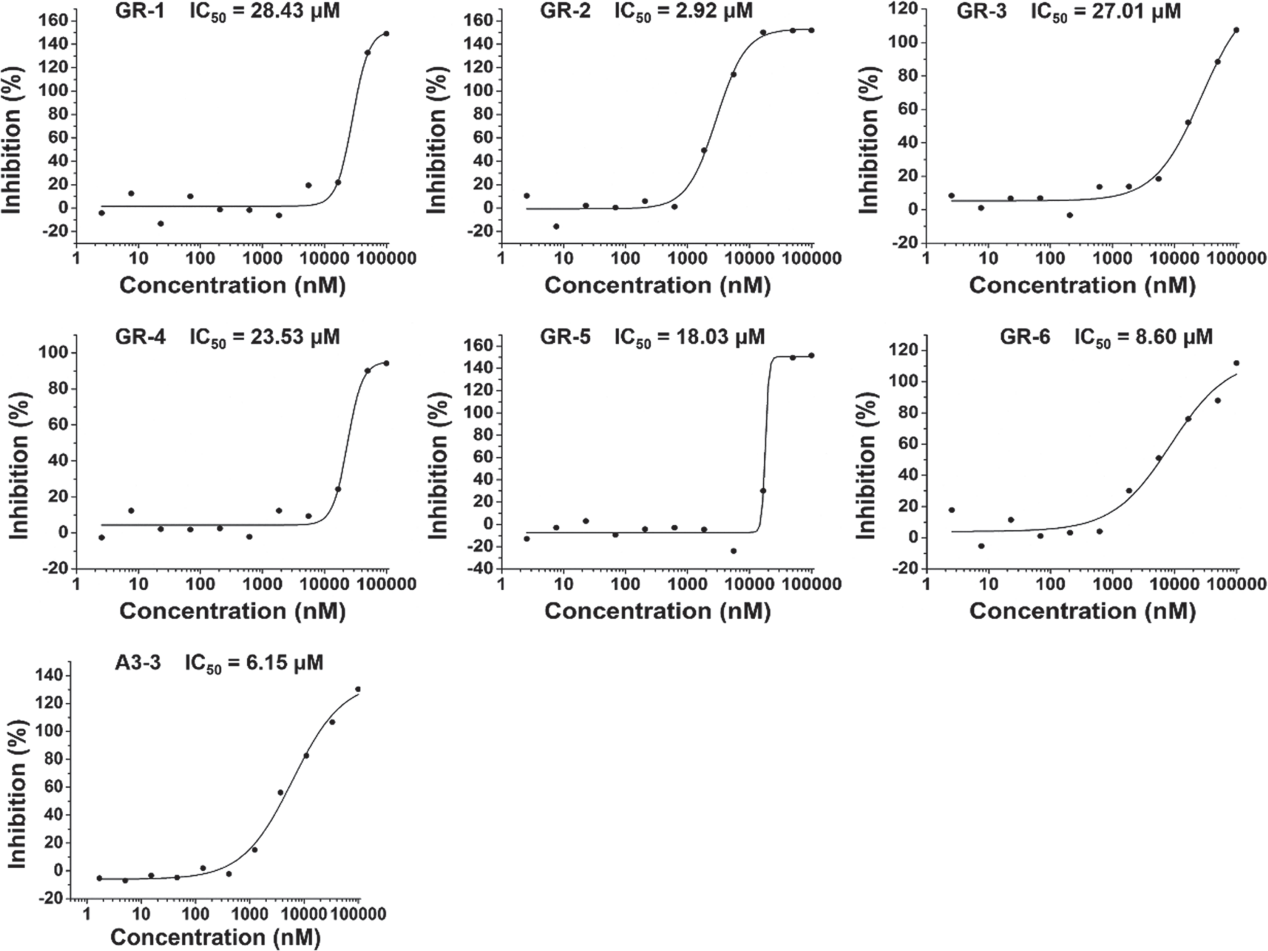
The binding curves for the predicted compounds in Table 1.

### Database content

GCDB contains 275 genes, 105 proteins, 40 approved drugs, 90 206 chemicals associated with 213 093 records of reported glaucoma bioactivities from 22 324 glaucoma corresponding bioassays and 5630 references. An overview of glaucoma-related proteins, drugs and signalling pathways involving glaucoma-related proteins and approved drugs is shown in Figure 3. A total of 105 glaucoma-related proteins can be grouped into the following categories (Figure 3A): (i) 51 enzymes such as carbonic anhydrase, LIM domain kinase, prostaglandin synthase and Rho-associated protein kinase; (ii) 21 ion channels such as sodium channel protein, voltage-gated calcium channel and transient receptor potential cation channel; (iii) 29 membrane receptors such as prostaglandin receptor, muscarinic acetylcholine receptor M3, adrenergic receptor, adenosine receptor (AR) and 5-hydroxytryptamine receptor; (iv) one secreted protein, VEGFA; (v) one transcription factor, the glucocorticoid receptor; and (vi) two transporters, norepinephrine transporter and solute carrier family 12 member 1.

A total of 40 marketed Drugs are used in the clinic for glaucoma treatment and 43 candidates are at various phases of clinical trials (Supplementary Table S1). In Figure 3B, glaucoma drugs in different stages of development are diagrammed according to their respective interacting targets. Among the glaucoma targets, ß1 adrenergic receptor has the largest number of marketed drugs, ß2 adrenergic receptor and ß3 adrenergic receptor have the second and third largest number of marketed drugs, respectively, which may be attributed to the fact that ß-adrenergic receptor antagonists can significantly reduce IOP with much fewer side effects than earlier glaucoma drugs (29). Following ß adrenergic receptors, Rho-associated protein kinases (ROCKs) have the highest number of drugs in development. With the recent development of new therapies, the role of eye tissues [e.g. trabecular meshwork (TM) (30) and optic nerve head (ONH) (31)] in glaucoma pathogenesis is better understood. ROCK inhibitors and AR ligands were found to play a role in modulating the pathways of extracellular matrix and cytoskeletal restructuring, not only lowering IOP and increasing ONH blood flow but also protecting against RGC loss (32). ROCK inhibitors based on isoquinoline and pyridine scaffolds show better potency and selectivity for ROCKs in treating glaucoma (33, 34). Currently, ROCKs as new therapeutic targets in late stage development have drawn much attention in glaucoma treatment due to multiple beneficial effects. At present, there are two drugs (Glanatec and Rhopressa) approved for the treatment of glaucoma, whereas six drugs targeting ROCKs have been discontinued due to safety, tolerability or insufficient efficacy during the Phase I and Phase II stages (35–37). Despite this, two ROCK inhibitors (PG324 and AMA0076) are in clinical trials and appear to be promising future for glaucoma therapeutics (38, 39). The benefits of neuroprotective activity and IOP reduction make these molecules a great potential tool for clinical treatment. Similarly, several AR ligands, which have neuroprotective benefits in addition to reducing IOP, as with ROCK inhibitors, are being developed in phases I, II and III clinical trials, as shown in Figure 3B.

GCDB also contains 677 drugs that are used to treat other diseases by regulating glaucoma-related targets, which indicates that glaucoma-related targets may be involved in several different pathways and are associated with the pathogenesis and development of various diseases. A total of 105 glaucoma-related protein targets and 40 marketed drugs were mapped onto the KEGG database to determine their corresponding pathways. The corresponding information on the signalling pathways of these targets and drugs were retrieved from the KEGG database. Figure 3C shows the pathways involving glaucoma targets and drugs. Neuroactive ligand–receptor interaction (KEGGID: hsa04080) has the highest number of glaucoma targets and drugs. This indicates that, in the long term, the emphasis of glaucoma treatment should focus on developing novel neuroprotective therapies. However, we did not observe pathways associated with immune cell activation, although it has been reported that early immune responses related to multiple genes and pathways are directly induced by elevated IOP (40). As shown in Figure 3C, multiple pathways play a critical role in the pathogenic mechanism and the treatment of glaucoma, and combinatorial targeting against multiple pathways could be more effective at inhibiting neurodegeneration in glaucoma (41). To the best of our knowledge, GCDB is the first comprehensive chemogenomics database for glaucoma. The data storage and retrieval in GCDB can facilitate *in silico* drug design. It needs to be emphasized that all the data in GCDB and associated information were collected from public databases, we cannot exclude the possibility that some of the data is erroneous, less specific or changing over time. The accuracy and completeness of the data in GCDB is dependent on the accuracy of data sources and the data processing operations (see Materials and methods section). Therefore, the results of GCDB need to be further analyzed and experimentally validated. The following case study demonstrates the application of GCDB in glaucoma drug research.

**Table 1:** New ligand-target prediction confirmed by *in vitro* experiment

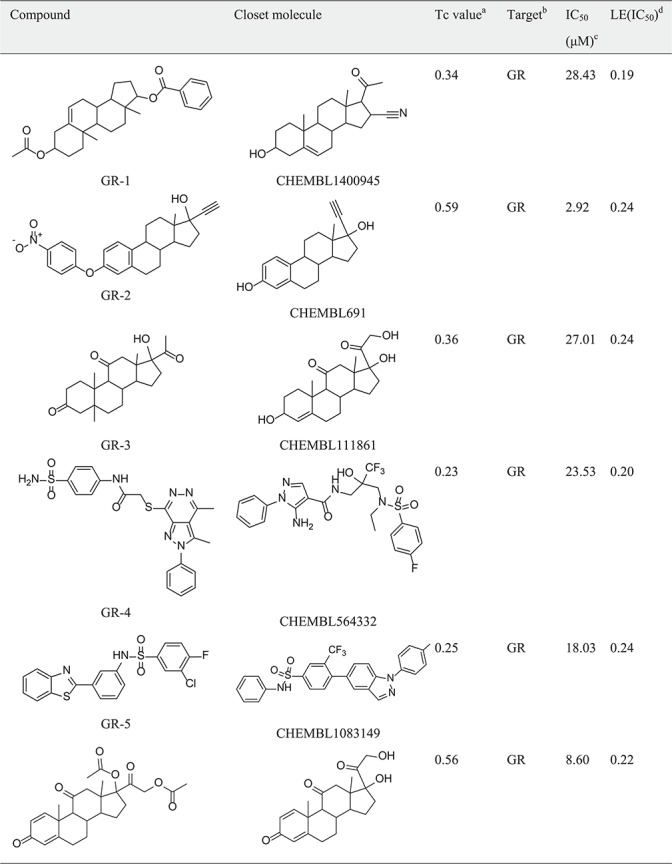

*(continued).*

**Table 1:** (continued).

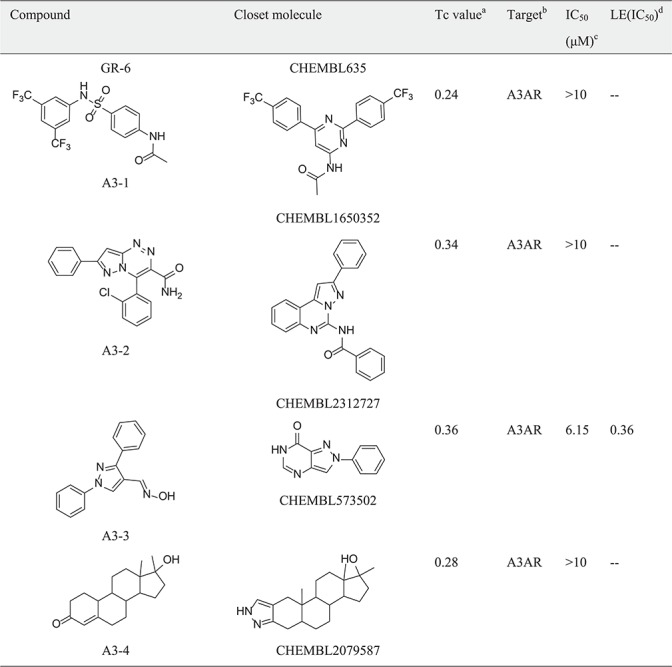

a ^a^Tc value: Similairty calculated between compounds predicted and the closest reference shown in the target set.

b ^b^Target: The predicted target from SEA predictions.

c ^c^IC_50_: Half-maximal inhibitory concentration.

d ^d^LE(IC_50_): (1.37 × pIC_50_)/HA, HA denotes the number of non-hydrogen atoms.

### Case study: identification of inhibitors of hGR and A3AR

Among the identified AR ligands, most AR, A2AAR and A3AR antagonists have shown potential for lowering IOP in clinical trials (30). Thus far, the Food and Drug Administration has not approved any drug targeting AR for treating glaucoma due to poor pharmacokinetic properties and bioavailability. Identification of new scaffolds via virtual screening or high-throughput screening will be helpful to design more potent inhibitors with better bioavailability in particular eye tissues and improve the effectiveness of glaucoma therapies. In addition, as shown in Figure 3B, hGR has two drugs in Phase II and one drug in Phase III trials, which are farther along in the clinical trial process compared with other targets. In the current clinical studies, drugs targeting hGR are used to speed healing after glaucoma surgery, further improving the treatment of glaucoma.

A total of 265 187 compounds from the Specs and Maybridge databases were computationally screened for their likelihood to bind to 105 targets using SEA training from GCDB. Then, 265 187 compounds were compared with each set of ligands for 105 targets related to glaucoma, yielding 72 612 drug-target comparisons. Among them, 490 and 4174 drug-target pairs involving hGR and A3AR, respectively, were considered in this study. The candidate compounds were filtered to be sufficiently different from known hGR and A3AR ligands. Large-scale Tanimoto coefficients (Tc) were calculated to estimate the degree of similarity between the candidate compounds and the compounds with known activity extracted from the ChEMBL and Binding databases using Extended-connectivity fingerprints (ECFP6) (42). Compounds with Tc values less than 0.6 were kept for further analysis (43). Most of the remaining compounds (447 compounds for hGR and 3938 compounds for A3AR) had no remarkable similarities to known ligands. To evaluate the variance in potential binding affinity, 447 and 3938 hits were docked into hGR and A3AR, respectively, by Glide SP to calculate their respective docking scores. Figure 4A shows a brief distribution of the docking scores, where the docking score is on the x-axis and the number of molecules in each docking score region is on the y-axis. Of note, the majority (∼90% for hGR and 75% for A3AR) of hits have a docking score better or equal to −6.0 kcal/mol (a more negative docking score indicates a better fit at the binding site). Moreover, to ensure chemical diversity, 447 and 3938 hits were imported into Canvas and clustered into 50 clusters according to their chemical similarity using Tanimoto similarity metric (44, 45). Figure 4B shows that the majority (∼70%) of the 447 hits clustered into cluster numbers 13, 16, 35, 36, 38, 39, 46 and 48. Figure 4C shows that majority (∼78%) of the 3938 hits were clustered into cluster numbers 13, 19, 24 and 33. Structurally diverse representatives from each cluster with the best docking scores and the strongest interactions with the key amino acids (details are reported in the Materials and methods section) were selected for analysis of *in vitro* biological activity. Finally, 10 compounds that were experimentally accessible to us were evaluated for *in vitro* biological activity, including six compounds for hGR and four compounds for A3AR. The top docking poses of six and four compounds in the binding pocket of hGR and A3AR, respectively, are shown in Supplementary Figures S1 and S2.

In the hGR reporter gene assay, most of selected compounds are highly potent for hGR. Of six test compounds, two yielded half-maximal inhibitory concentration (IC_50_) values lower (better) than 10 μM derived from the concentration–response curves (Figure 5), and the remainder had an IC_50_ value better than 30 μM (Table 1). Diversity of the identified hGR ligands are shown visually in Table 1. The inhibition potency of the four selected compounds was evaluated based on the cAMP level of ADORA3-CHO cells elicited by NECA. One of these bound to A3AR with an IC_50_ value better than 10 μM. The binding curve of the most efficient A3AR binder is presented in Figure 5. The identified ligands of hGR and A3AR shown in Table 1 have ligand efficiencies (LEs) based on IC_50_ values ∼0.2 kcal/mol per heavy atom, which provides more space for structure optimisation. LE is considered to be a good metric for optimisation based on molecular mass to generate high-activity lead-like compounds (46). Compound A3-3 presented the highest LE value, at 0.36 kcal/mol, which consists of 20 heavy atoms with a molecular weight of 263. In addition, GR-4 and GR-5 represent novel, non-steroidal hGR ligands, which make them attractive candidates to explore for future therapeutic applications. A key question in new predictions is whether the scaffolds are novel. To evaluate the novelty of new predictions, we compared the similarity between hits and the previously known ligands in hGR and A3AR target sets. The Tc was used to compare the level of similarity between two molecules based on an ECFP6 fingerprint (42). The closest known ligands with the highest chemical similarity (Tc value) to the hits are listed in Table 1. The results in Table 1 show that four of the six tested hGR binders are weak inhibitors of hGR in an *in vitro* inhibitory assay, with IC_50_ values ranging from 18.03–28.43 μM, and the Tc values of these hits are all less than 0.4. Moreover, compound GR-2 and compound GR-6 have IC_50_ values of 2.92 and 8.60 μM, respectively, and Tc values greater than 0.5. This indicates that the more similar in structure, the more likely new identified hits share the same targets with the closest known ligands. It should be noted that compound A3-3 was the most efficient A3AR binder, with an IC_50_ better than 10 μM and a Tc value less than 0.4 to the closest known A3AR ligand. Despite low similarity to the closest known A3AR ligand, the newly identified hit from SEA prediction is a promising candidate for A3AR, with an IC_50_ value of 6.15 μM. In summary, of the 10 predictions that were assayed, 70% were confirmed to be active. The screening study resulted in five out of seven hits with a Tanimoto similarity value less than 0.4 to known ligands, which illustrates the novelty of chemical structures. The screening strategy that we used could be applied to other glaucoma targets of interest to design novel chemical scaffolds.

Currently, research into biological processes requires comprehensive consideration of the biological system rather than specific biological instances. This requires that researchers focus on systemisation and summarisation of relevant biomedical information of biological instances from the literature and link to specified database entries. This process is labour intensive and it is difficult to ensure the quality and integrity of the data. Therefore, it is necessary to develop new methods and tools to save time and effort spent on organising instances and to reduce the number of repetitive tasks. GCDB is a specific database on glaucoma that organizes all drugs and protein targets related to glaucoma. GCDB has a special range of features and capabilities. For instance, a structure similarity search in the GCDB database can be used to rapidly search various compound databases for relevant glaucoma-related proteins without requiring any user input. Moreover, the cheminformatics tool (SEA) can be used for the discovery of lead compounds for novel strategies specialized for glaucoma treatment. By integrating many glaucoma-related drugs and small chemical moieties with target annotations, GCDB offers specific data and tool to help researchers gain insight into the significance of glaucoma-related targets and drugs. This will improve efficiency and productivity in the drug discovery and design process.

## Conclusion

With the accumulation of biological data, a more specific database can dramatically improve the efficiency of drug discovery. Liu *et al.* developed an integrated cloud computing server, *AlzPlatform*, focusing on assembling chemogenomics data associated with Alzheimer’s disease (AD) and providing multiple online computing programs for target identification, drug repurposing and polypharmacology analysis (47). AlzPlatform has been proven to be a valuable platform for the prediction of potential novel AD drug targets and screening new AD-active molecules by bioactivity and *in vitro* experimental validation. GCDB is currently the only platform available for the identification of potential drug targets and for screening new drug scaffolds for glaucoma treatment. The glaucoma database website allows analysis of the pharmacological target space and searches for drugs and targets of interest. The platform also provides implementation of a ligand-based computational algorithm for virtual screening and target prediction by assessing the activity of a query molecule based on its similarity to active ligands of a given target. In addition, GCDB contributes to the discovery of multi-target-directed ligands to combat glaucoma through systematic prediction of chemical–protein interactions for approved anti-glaucoma drugs and known active compounds related to glaucoma. GCDB is helpful to understand the mechanisms of disease progression as well as repurposing drugs. For this version of the database, our cheminformatics tool has certain limitations, including a deficiency of ligands for a certain targets. We will continue to update the related data weekly. Additionally, we will implement other powerful computational algorithms as complementary partners, like the structure-based tool. Many drug design tools will be updated to the database including ADMET, molecular docking and gene ontology tools. We will build a comprehensive platform on glaucoma to further help in drug discovery of glaucoma treatment. In short, this study showed that the improved chemical SEA has been successfully used to identify new glaucoma chemical molecules, as verified by *in vitro* experiments. We hope that GCDB will facilitate research into new drugs and treatments for glaucoma.

## Supplementary Material

Supplementary Data

## Notes

**Database URL:** http://cadd.pharmacy.nankai.edu.cn/gcdb/home
