# Supplementary material for: GCDB: a glaucomatous chemogenomics database for in silico drug discovery
Source: Database (Oxford). 2018 Oct 29;2018:bay117. doi: 10.1093/database/bay117 (PMC6204718; doi:10.1093/database/bay117)
Supplement: Supplementary Data [file bay117_supp.zip › EDITORIAL CERTIFICATE.pdf]

ACS ChemWorx AUTHORING SERVICES

EDITORIAL CERTIFICATE

This document certifies that the manuscript listed below was edited for proper English language, grammar, punctuation, spelling, and overall style by one or more of the highly qualified native English speaking editors at ACS ChemWorx Authoring Services.

MANUSCRIPT TITLE:

GCDB: A Glaucomatous Chemogenomics Database for in silico Drug Discovery

AUTHORS:

Yu Wei, Jinlong Li, Baiqing Li, Chunfeng Ma, Xuanming Xu, Xu Wang, Aqin Liu, Tengfei Du, Zhonghua Wang, Zhangyong Hong, Jianping Lin

DATE ISSUED:

July 13, 2018

CERTIFICATE VERIFICATION KEY:

F02C-6766-14B5-6325-DE83

This certificate may be verified at secure.es.acschemworx.acs.org/certificate. This document certifies that the manuscript listed above was edited for proper English language, grammar, punctuation, spelling, and overall style by highly qualified native English speaking editors at ACS ChemWorx AuS. Neither the research content nor the authors' intentions were altered in any way during the editing process. Documents receiving this certification should be English-ready for publication; however, the author is able to accept or reject our suggestions and changes. To verify the final ACS ChemWorx AuS edited version, please visit our verification page. If you have any questions or concerns about this edited document, please contact ACS ChemWorx AuS at support@es.acschemworx.acs.org. AUTHORS: Please use caution when sharing your certificate verification code. Anyone you provide this code can download the final version of the ACS ChemWorx AuS manuscript. Please note that this certificate DOES NOT guarantee manuscript acceptance.

ACS ChemWorx Authoring Services provides editing services for researchers around the world. Our top-quality editors are all native English speakers from America's top universities. Our editors come from nearly every research field and possess the highest qualifications to edit research manuscripts written by non-native English speakers. For more information about our services, please visit es.acschemworx.acs.org.
