# Supplementary material for: GCDB: a glaucomatous chemogenomics database for in silico drug discovery
Source: Database (Oxford). 2018 Oct 29;2018:bay117. doi: 10.1093/database/bay117 (PMC6204718; doi:10.1093/database/bay117)
Supplement: Supplementary Data [file bay117_supp.zip › supporting information.docx]

**Supplementary material**

## GCDB: A Glaucomatous Chemogenomics Database for *in silico* Drug Discovery

**Yu Wei^a^, Jinlong Li^c^, Baiqing Li^a^, Chunfeng Ma^d^, Xuanming Xu^a^, Aqin Liu^a^, Tengfei Du^a^, Zhonghua Wang^c*^, Zhangyong Hong^b*^, Jianping Lin ^a,c,d*^**

^a^State Key Laboratory of Medicinal Chemical Biology, College of Pharmacy and Tianjin Key Laboratory of Molecular Drug Research, Nankai University, Haihe Education Park, 38 Tongyan Road, Tianjin 300353, China

^b^State Key Laboratory of Medicinal Chemical Biology, College of Life Sciences, Nankai University, 94 Weijin Road, Tianjin 300071, China

^c^Biodesign Center, Tianjin Institute of Industrial Biotechnology, Chinese Academy of Sciences, Tianjin 300308, China

^d^Platform of Pharmaceutical Intelligence, Tianjin International Joint Academy of Biomedicine, Tianjin, 300000, China

*Corresponding authors:

Jianping Lin, State Key Laboratory of Medicinal Chemical Biology, College of Pharmacy and Tianjin Key Laboratory of Molecular Drug Research, Nankai University, Haihe Education Park, 38 Tongyan Road, Tianjin 300353, China, email: [jianpinglin@nankai.edu.cn](mailto:jianpinglin@nankai.edu.cn)

Zhangyong Hong, College of Life Sciences, Nankai University, 94 Weijin Road, Tianjin 300071, China, email: [hongzy@nankai.edu.cn](mailto:hongzy@nankai.edu.cn)

Zhonghua Wang, Biodesign Center, Tianjin Institute of Industrial Biotechnology, Chinese Academy of Sciences, Tianjin 300308, China, email: [wang_zh@tib.cas.cn](mailto:wang_zh@tib.cas.cn)

**Contents:**

**Table S1.** 40 marketed and 43 clinical trial drugs against glaucoma.

**Figure S1.** Top binding poses of compounds GR-1, GR-2, GR-3, GR-4, GR-5 and GR-6 in the binding pocket of hGR. The structure of hGR is represented in cartoon style. Key residues in the binding pocket are shown in lines. Compounds GR-1, GR-2, GR-3, GR-4, GR-5 and GR-6 are shown in sticks. Potential hydrogen bonding interaction are shown as red dashed lines.

**Figure S2.** Top binding poses of compounds A3-1, A3-2, A3-3 and A3-4 in the binding pocket of A3AR. A3AR is represented in cartoon style. Key residues in the binding pocket are shown in lines. Compounds A3-1, A3-2, A3-3 and A3-4 are shown in sticks. Potential hydrogen bonding interaction are shown as red dashed lines.

**Table S1. 40 marketed and 43** **clinical trial drugs against glaucoma.**

|  | **Drug for Glaucoma treatment** | **Drug state** | **Reference** |
| --- | --- | --- | --- |
| 1 | AC-262271 | Phase 1 | http://en.pharmacodia.com/web/drug/1_22084.html |
| 2 | ACECLIDINE | Approved | http://www.kegg.jp/entry/D02750 |
| 3 | Acetazolamide | Approved | http://www.kegg.jp/entry/D00218 |
| 4 | Adaprolol maleate | Discontinued in Phase 2 | http://en.pharmacodia.com/web/drug/1_8830.html |
| 5 | ALO-1567 | Discontinued in Phase 1 | https://www.pharmacodia.com/web/drug/q_8_923.html |
| 6 | ALPRENOXIME HYDROCHLORIDE | Discontinued in Phase 2 | http://en.pharmacodia.com/web/drug/1_8864.html |
| 7 | AMA0076 | Phase 2 | http://glaucomatoday.com/2012/12/phase-2a-trial-of-rock-inhibitor-initiated |
| 8 | Apraclonidine | Approved | http://www.kegg.jp/entry/D07461 |
| 9 | AR-102 | Phase 2 | https://www.clinicaltrials.gov/ct2/show/NCT00523250?cond=AR-102&rank=1 |
| 10 | AR-12286 | Discontinued in Phase 2 | https://clinicaltrials.gov/ct2/show/NCT01330979?cond=AR-12286&rank=2 |
| 11 | ATS907 | Discontinued | https://clinicaltrials.gov/ct2/show/NCT01668524?cond=ATS907&rank=1 |
| 12 | Befunolol hci | Approved | http://www.kegg.jp/entry/D07496 |
| 13 | Betamethasone | Phase 2 | https://clinicaltrials.gov/ct2/show/NCT02114073?cond=Betamethasone+glaucoma&rank=1 |
| 14 | Betaxolol hydrochloride (JP17/USP) | Approved | http://www.kegg.jp/entry/D07526 |
| 15 | BHT-920 | Approved | http://bidd.nus.edu.sg/BIDD-Databases/TTD/ZFTTDDRUG.asp?ID=DNC000312 |
| 16 | Bimatoprost | Approved | http://www.kegg.jp/entry/D02724 |
| 17 | bosentan | Phase 1 | https://clinicaltrials.gov/ct2/show/NCT00701597 |
| 18 | Brimonidine | Approved | https://www.drugbank.ca/drugs/DB00484 |
| 19 | Brinzolamide | Approved | https://www.drugbank.ca/drugs/DB01194 |
| 20 | Bunazosin | Approved | http://www.kegg.jp/dbget-bin/www_bget?dr:D01887 |
| 21 | Bupranolol | Approved | https://www.drugbank.ca/drugs/DB08808 |
| 22 | BVT.28949 | Phase 2 | http://bidd.nus.edu.sg/BIDD-Databases/TTD/ZFTTDDRUG.asp?ID=DIB015491 |
| 23 | Carbachol | Approved | https://www.drugbank.ca/drugs/DB00411 |
| 24 | Carteolol | Approved | https://www.drugbank.ca/drugs/DB00521 |
| 25 | DE-104 | Discontinued in Phase 2 | http://en.pharmacodia.com/web/drug/1_3346.html |
| 26 | DE-117 | Phase 3 | https://clinicaltrials.gov/ct2/show/NCT02981446 |
| 27 | Demecarium | Approved | https://www.drugbank.ca/drugs/DB00944 |
| 28 | Dexamethasone | Phase 3 | https://clinicaltrials.gov/ct2/show/NCT01228149?cond=Dexamethasone+glaucoma&rank=3 |
| 29 | Dichlorphenamide | Approved | https://www.drugbank.ca/drugs/DB01144 |
| 30 | Diclofenac | Phase 4(Phase 3) | https://clinicaltrials.gov/ct2/show/NCT00825864?cond=Diclofenac+glaucoma&rank=1 |
| 31 | Dipivefrin | Approved | https://www.drugbank.ca/drugs/DB00449 |
| 32 | Distigmine bromide | Approved | https://www.pharmacodia.com/web/drug/1_2126.html |
| 33 | Dorzolamide | Approved | https://www.drugbank.ca/drugs/DB00869 |
| 34 | Echothiophate iodide | Approved | https://www.drugbank.ca/drugs/DB01057 |
| 35 | Enalkiren | Discontinued in Phase 2 | http://bidd.nus.edu.sg/BIDD-Databases/TTD/ZFTTDDRUG.asp?ID=DNC000604 |
| 36 | Epinephrine | Approved | https://www.drugbank.ca/drugs/DB00668 |
| 37 | Ethacrynic acid | Phase 2 | Invest. Ophthalmol. Vis. Sci. 2005, 46: 3680 |
| 38 | Ethoxzolamide | Withdrawn from the Market | https://www.drugbank.ca/drugs/DB00311 |
| 39 | Evodenoson （DE-112） | Discontinued in Phase 2 | http://en.pharmacodia.com/web/drug/1_13406.html |
| 40 | Glycerin | Approved | http://www.kegg.jp/dbget-bin/www_bget?dr:D00028 |
| 41 | INO-8875 (Trabodenoson) | Discontinue in Phase 3 | https://www.epgonline.org/global/news/trabodenoson--ino-8875--fails-phase-iii-trial-for-primary-open-angle-glaucoma---inotek-pharmaceuticals-corporation-.html |
| 42 | INS115644 | Discontinued in Phase 1 | Invest. Ophthalmol. Vis. Sci. 2010; 51(13):6432. |
| 43 | INS117548 | Discontinued in Phase 1 | http://en.pharmacodia.com/web/drug/1_26686.html |
| 44 | Isoflurophate | Approved | https://www.drugbank.ca/drugs/DB00677 |
| 45 | Isosorbide | Approved | http://www.kegg.jp/dbget-bin/www_bget?dr:D00347 |
| 46 | Latanoprost | Approved | https://www.drugbank.ca/drugs/DB00654 |
| 47 | Levobetaxolol | Approved | https://www.drugbank.ca/drugs/DB09351 |
| 48 | Levobunolol | Approved | https://www.drugbank.ca/drugs/DB01210 |
| 49 | LX7101 | Phase 2 | http://en.pharmacodia.com/web/drug/1_3290.html |
| 50 | Mannitol | Approved | http://www.kegg.jp/dbget-bin/www_bget?dr:D00062 |
| 51 | Memantine | Phase 3 | https://clinicaltrials.gov/ct2/show/NCT00168350 |
| 52 | Methazolamide | Approved | https://www.drugbank.ca/drugs/DB00703 |
| 53 | Metipranolol | Approved | https://www.drugbank.ca/drugs/DB01214 |
| 54 | Mitomycin | Approved | http://www.kegg.jp/dbget-bin/www_bget?dr:D00208 |
| 55 | moxaverine | Phase 3 | https://clinicaltrials.gov/ct2/show/NCT00709449?cond=moxaverine+glaucoma&rank=1 |
| 56 | Nipradilol | Approved | http://www.kegg.jp/dbget-bin/www_bget?dr:D01691 |
| 57 | ONO-9054 | Phase 2 | https://clinicaltrials.gov/ct2/show/NCT02083289?cond=ONO-9054+glaucoma&rank=1 |
| 58 | OPA-6566 | Phase 2 | https://clinicaltrials.gov/ct2/show/NCT01410188?cond=OPA-6566+glaucoma&rank=1 |
| 59 | PBF-677 | Phase 1 | http://en.pharmacodia.com/web/drug/1_13404.html |
| 60 | PG324(Roclatan) | Phase 3 | https://clinicaltrials.gov/ct2/show/NCT02674854 |
| 61 | PGF2ALPHA-IE | Phase 3 | http://bidd.nus.edu.sg/BIDD-Databases/TTD/ZFTTDDRUG.asp?ID=DIB002812 |
| 62 | Physostigmine | Approved | https://www.drugbank.ca/drugs/DB00981 |
| 63 | Piclidenoson (CF101) | Phase 3 | http://en.pharmacodia.com/web/drug/1_1864.html |
| 64 | Pilocarpine | Approved | https://www.drugbank.ca/drugs/DB01085 |
| 65 | PROXODOLOL | Discontinued in Phase 2 | http://en.pharmacodia.com/web/drug/1_5872.html |
| 66 | QLT-091568 | Discontinued in Phase 2 | http://en.pharmacodia.com/web/drug/1_8734.html |
| 67 | QPI-1007 | Phase 2/3 (Phase 3) | https://clinicaltrials.gov/ct2/show/NCT01965106?cond=QPI-1007+glaucoma&rank=1 |
| 68 | Rhopressa （Netarsudil） | Approved | https://www.drugbank.ca/drugs/DB13931 |
| 69 | Ripasudil | Approved | https://www.drugbank.ca/drugs/DB13165 |
| 70 | RO5093151 | Phase 1 | https://clinicaltrials.gov/ct2/show/NCT02622334 |
| 71 | SAR366234 | Discontinued in Phase 1 | http://en.pharmacodia.com/web/drug/1_3960.html |
| 72 | SDZ-GLC-756 | Discontinued in Phase 1 | http://en.pharmacodia.com/web/drug/1_12744.html |
| 73 | Sezolamide hydrochloride | Approved | http://www.genome.jp/dbget-bin/www_bget?D03845 |
| 74 | Simenepag | Approved | http://www.kegg.jp/dbget-bin/www_bget?dr:D09962 |
| 75 | SR-43845 | Phase 2 | https://db.idrblab.org/ttd/drug/d0h8wz |
| 76 | SYL040012 | Phase 2 | Invest. Ophthalmol. Vis. Sci. 2014, 55: 564 |
| 77 | Tafluprost | Approved | https://www.drugbank.ca/drugs/DB08819 |
| 78 | Taprenepag | Discontinued in Phase 2 | http://en.pharmacodia.com/web/drug/1_22264.html |
| 79 | Timolol | Approved | https://www.drugbank.ca/drugs/DB00373 |
| 80 | Travoprost | Approved | https://www.drugbank.ca/drugs/DB00287 |
| 81 | Triamcinolone | Phase 2 | https://clinicaltrials.gov/ct2/show/NCT00853905?cond=Triamcinolone+glaucoma&rank=4 |
| 82 | Unoprostone | Discontinued | https://www.drugbank.ca/drugs/DB06826 |
| 83 | Y39983 | Discontinued in Phase 2 | http://en.pharmacodia.com/web/drug/1_1139.html |


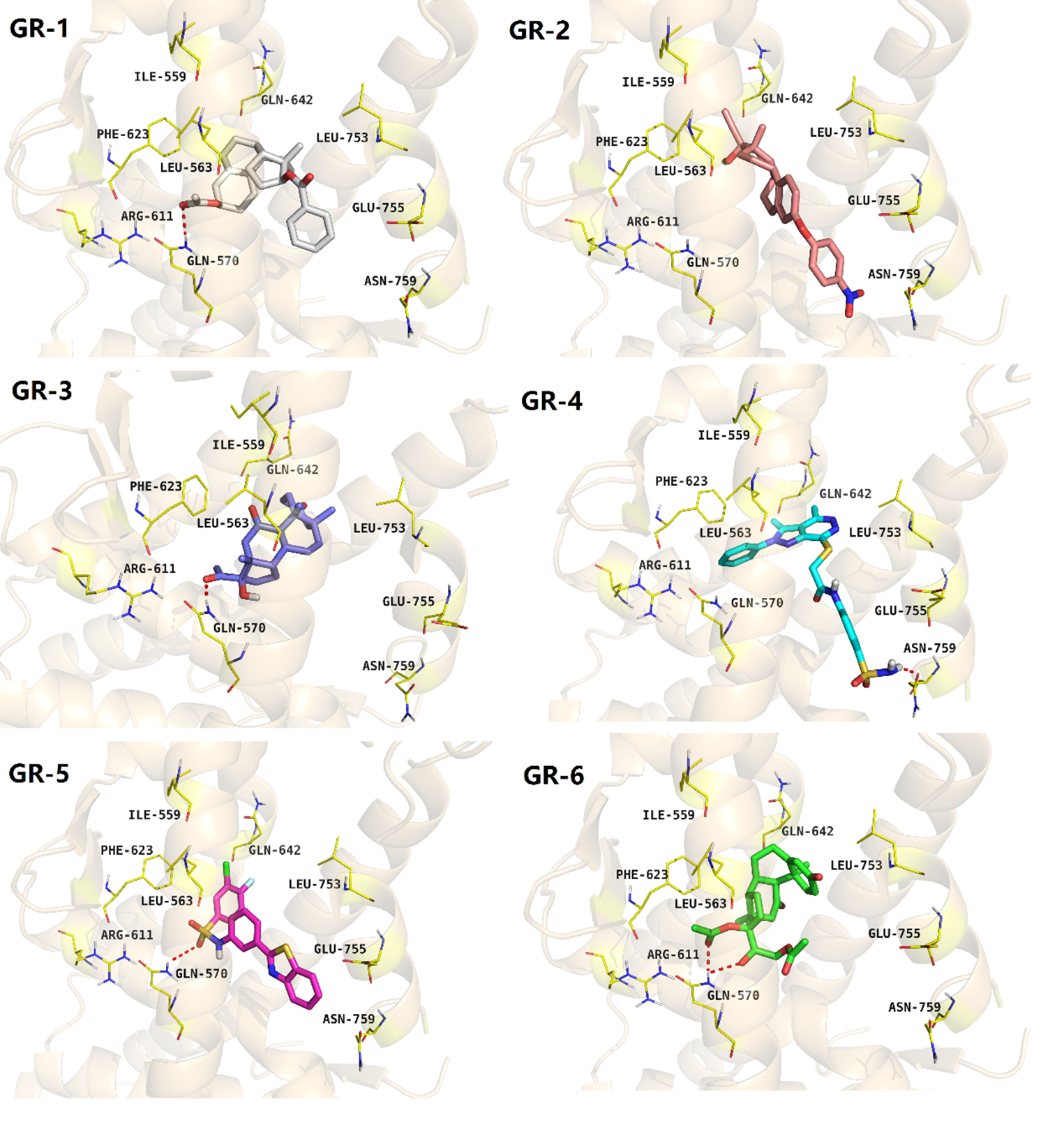


**Figure S1.** Top binding poses of compounds GR-1, GR-2, GR-3, GR-4, GR-5 and GR-6 in the binding pocket of hGR. The structure of hGR is represented in cartoon style. Key residues in the binding pocket are shown in lines. Compounds GR-1, GR-2, GR-3, GR-4, GR-5 and GR-6 are shown in sticks. Potential hydrogen bonding interaction are shown as red dashed lines.


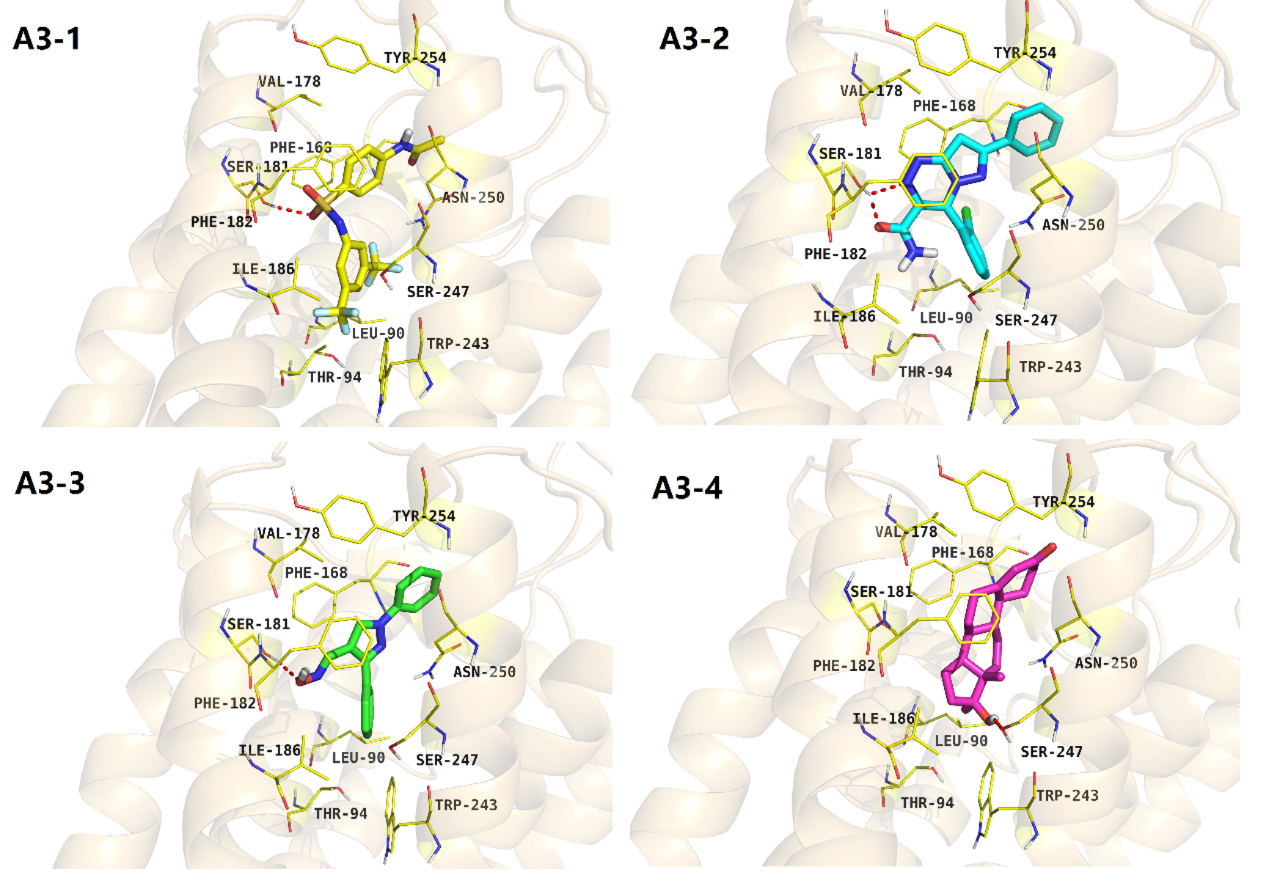


**Figure S2.** Top binding poses of compounds A3-1, A3-2, A3-3 and A3-4 in the binding pocket of A3AR. A3AR is represented in cartoon style. Key residues in the binding pocket are shown in lines. Compounds A3-1, A3-2, A3-3 and A3-4 are shown in sticks. Potential hydrogen bonding interaction are shown as red dashed lines.
